# Idiopathic Intracranial Hypertension After Abrupt Cessation of Medication: A Case Report of Abrupt Glucagon-Like Peptide-1 (GLP-1) Receptor Agonist Cessation and Review of the Literature

**DOI:** 10.1007/s11916-024-01215-9

**Published:** 2024-04-04

**Authors:** Brittany Heckel

**Affiliations:** Jefferson Headache Center, Department of Neurology, Thomas Jefferson University, Philadelphia, PA USA

**Keywords:** Idiopathic intracranial hypertension, Glucagon-like peptide-1 (GLP-1) agonists, Obesity, Duraglutide, Pseudotumor cerebri

## Abstract

**Purpose of Review:**

The purpose of this review is two-fold: (1) to discuss a case report of idiopathic intracranial hypertension (IIH) after abrupt cessation of a glucagon-like peptide-1 (GLP-1) receptor agonist with resultant rapid weight gain and (2) to review the literature regarding the potential role of GLP-1 receptor agonists in the treatment of IIH as well as potential pitfalls.

**Recent Findings:**

GLP-1 receptor agonists have become widely used to treat obesity. Obesity is a known risk factor for the development of IIH, though the precise pathophysiology is unclear. GLP-1 receptor agonists may help treat IIH by promoting weight loss, lipolysis of adipose tissue, and potentially decreasing the secretion of CSF, as was seen in rat models. Abrupt cessation of GLP-1 receptor agonists can result in regaining lost weight rapidly. In the case that we present, the patient stopped duraglutide abruptly due to lack of insurance coverage and regained the weight she had lost within a month. She subsequently developed IIH.

**Summary:**

GLP-1 receptor agonists have the potential to help treat IIH; however, this class of medication needs to be used carefully, as cessation of the medication and resultant rapid weight gain can result in IIH.

## Introduction

**Fig. 1:**
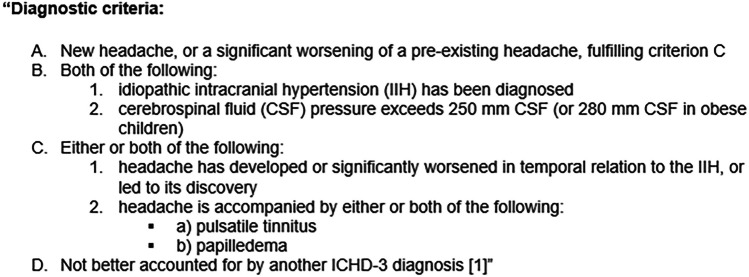
Diagnostic criteria for idiopathic intracranial hypertension (IIH), per “The International Classification of Headache Disorders, Third Edition (ICHD-3) [1]”

Idiopathic intracranial hypertension (IIH) is a type of secondary headache disorder characterized by increased intracranial pressure; as the name implies, the precise etiology of the increased pressure in IIH is unknown. “The International Classification of Headache Disorders, Third Edition (ICHD-3),” provides the diagnostic criteria for this disorder (Fig. 1) [1].

IIH occurs predominantly in women of childbearing age who have obesity [2]. Over 90% of individuals with IIH are obese [3•]. There is also an association with the development of IIH and rapid weight gain [2, 4]. As the rate of obesity has increased, there has been a corresponding increase in the incidence and the prevalence of IIH, with a population-based matched controlled cohort study in the UK showing both the incidence and the prevalence of IIH in women more than tripling from 2005 to 2017 [5]. Weight loss of between 3 and 15% of body weight at initial diagnosis has been associated with remission of IIH [6, 7••, 8]. Despite the association between obesity and IIH, there have been few prospective studies looking at the precise interaction between weight loss and IIH, leading to a dearth of evidence-based therapeutic guidelines [8].

With the increasing recognition of obesity as a complex disease requiring active medical management and the increasing popularity of effective drugs for weight loss, such as glucagon-like peptide-1 (GLP-1) receptor agonists, which include medications such as tirzepatide (Mounjaro®, Eli Lilly, Indianapolis, IN), liraglutide (Victoza®, Novo Nordisk, Plainsboro, NJ; and Saxenda ®, Novo Nordisk, Plainsboro, NJ), dulaglutide (Trulicity®, Eli Lilly, Indianapolis, IN), and semaglutide (Ozempic®, Novo Nordisk, Plainsboro, NJ), there is burgeoning potential to induce IIH remission in the setting of use of these medications [9]. The weight loss, however, would need to be maintained to achieve the desired remission, as small studies have also shown the recurrence of IIH when weight is regained after weight loss [9]. This article discusses a case of a patient who developed IIH after the abrupt cessation of a GLP-1 receptor agonist with resultant rapid weight gain and discusses the potential ways that the GLP-1 receptor agonists may be useful in the treatment of IIH.

## Case

### Clinical Presentation

The female patient initially presented to our clinic at age 27 for evaluation of roughly 1 year of more severe and frequent headache. The patient had a past medical history significant for May-Thurner Syndrome with iliac stent placement on anticoagulation, anxiety, and obesity (body mass index (BMI) of 34 kg/m^2^). As far as her headache history, the patient reported episodic migraine beginning at age 12. The patient reported having an unremarkable magnetic resonance imaging (MRI) scan of the brain in the past. Her headache improved as she reached adulthood, and she reported sometimes going for years without a headache. She had never been on a headache preventive.

Two years prior to presentation, the patient started treatment for obesity with duraglutide, a glucagon-like peptide-1 (GLP-1) receptor agonist. She remained on this treatment for roughly 1 year and 1 month, reportedly losing almost 30 pounds (BMI at that time would have been roughly 29 kg/m^2^.). Due to issues with insurance coverage, the patient stopped following with her prescribing physician and stopped the duraglutide. She reports she rapidly regained 30 pounds over the course of 1–2 months, and she returned to the weight she was at the initiation of the medication.

Within 1 month of stopping the duraglutide, her headache began to worsen. She went from only having an occasional headache to a daily headache. Headache pain was described as 6/10 in severity. She described the character of her pain as a pressure at the base of her skull and stabbing pain behind her eyes. She noted associated symptoms of nausea, emesis, photophobia, phonophobia, and lightheadedness. She also reported that her headache worsens with Valsalva. Two months after stopping the duraglutide, she reported that she saw an eye doctor for a dilated eye exam, and the patient reported that papilledema of the right optic nerve was noted. Due to continued issues with insurance and a move, she did not present to our headache center for a further 11 months.

Her examination in the office was notable for Grade I papilledema of the optic discs. Her neurologic exam was otherwise unremarkable.

### Diagnostic Studies

MRI of the brain showed stigmata of IIH, with a partially empty sella, prominence of Meckel’s cave, and prominence of cerebrospinal fluid (CSF) around the optic nerves. Magnetic resonance venography (MRV) showed hypoplastic left transverse and sigmoid sinuses. MRV also showed mild narrowing in the lateral part of the right transverse sinus. Lumbar puncture under fluoroscopy showed an elevated opening pressure of 27 cm of water with a closing pressure of 7 cm of water after draining 20 mL of CSF.

### Management/Treatment and Follow-Up

The patient improved significantly after lumbar puncture. She follows closely with ophthalmology for monitoring of her optic nerves and peripheral vision. She was initially started on topiramate, with significant improvement in her headache. After several months on topiramate, her headache again became more frequent, and she was started on acetazolamide, which provided an even more significant reduction in her headache severity and frequency. She currently has less than 15 headache days per month. She has recently started following with weight management for more sustained weight loss, working with a nutritionist.

## Discussion and Review of Recent Literature

In the case of our patient, it appears that she developed IIH due to the abrupt cessation of the GLP-1 receptor agonist prescribed to her with resultant rapid weight gain, with her BMI increasing from 29 to 34 in a little over a month. She did not previously have any features of IIH, and she had brain imaging without stigmata of IIH. Additionally, it is interesting that she did not present with IIH previously when at a BMI of 34 but only developed the condition when the weight was regained over a short period of time instead of the gradual build up over the years that led to her initially developing obesity.

### Obesity and IIH

As mentioned before, the precise pathophysiology of IIH has yet to be elucidated. Various theories postulate that IIH is either a problem of CSF overproduction or a result of difficulty with CSF drainage or absorption, but no unifying theory exists to fully explain the exact mechanism by which a patient develops IIH or the causation underlying the issues with CSF dynamics [9]. There is similarly limited information on why only certain individuals with obesity develop IIH [9]. A study using dual-energy X-ray absorptiometry (DEXA) scanning to determine if there was a difference in adipose distribution among patients with IIH and obesity and those without IIH and obesity showed a correlation between increased truncal adiposity and increased CSF pressure [3•]. The exact mechanism of this increase in CSF pressure is unknown, but it is postulated that increased truncal adiposity ultimately increases central venous pressure, though this mechanism still does not completely explain this phenomenon [10]. Weight loss with resultant loss of truncal adiposity correlated with decreased CSF pressure [3•]. Women with IIH also have twice the risk of cardiovascular disease than those of similar weight without IIH, but again, the pathophysiology underlying this is still not known [5].

The rapid rate of weight gain has also been implicated in the development of IIH. A study by Ko et al. showed that individuals who have recurrent IIH after weight loss and resolution gained back approximately 6% of their body weight and had an average rate of BMI gain of 1.3 kg/m(2)/year [11]. There is also thought to be a correlation with the rate of weight gain and IIH, with a case series showing women who rapidly gained weight within several months of receiving an implantable contraceptive developed IIH [10]. The women recovered with medication and weight loss while still having the implantable contraceptive device in place, suggesting that the etiology of the IIH was the rapidity of the weight gain, to which the implantable contraceptive device may have contributed [10].

Additionally, adipose tissue functions as an endocrine organ; proteins and hormones secreted by adipose tissue may also play a role in the development of IIH [9]. Leptin is a hormone which helps to regulate satiety in the hypothalamus; leptin receptors are located in the choroid plexus, where CSF is also produced [9]. Patients with IIH have increased leptin levels in the CSF, whereas other patients with obesity (but without IIH) have decreased leptin levels in the CSF [9]. The significance of this finding remains unclear, but it does remind us that adipose tissue secretes proteins and hormones which may be involved in the pathogenesis of IIH.

### GLP-1 Receptor Agonists and IIH

GLP-1 is a peptide hormone, an incretin, which is typically secreted by the distal small intestine after ingesting food [7••]. GLP-1 is involved in multiple processes, including the secretion of insulin, delaying gastric emptying, lipolysis of adipose tissue, increasing satiety through interactions with the hypothalamus, and decreasing CSF production in the choroid plexus [7••]. Capitalizing off of GLP-1’s appetite suppressing and metabolic effects, multiple GLP-1 receptor agonists are currently available for the treatment of obesity, as well as Type II diabetes mellitus. As GLP-1 receptor agonists decrease weight and promote lipolysis of adipose tissues, these medications could help treat IIH through weight loss and reduction in hormone-secreting adipose tissue. Recent studies in rat models have shown that GLP-1 receptor agonists decreased the CSF production and the intracranial pressure in rats [12••]. While further study is needed in humans, this could be another possible mechanism for GLP-1 receptor agonists to treat IIH. The risk of using GLP-1 receptor agonists to treat IIH seems to lie primarily in the potential for individuals to rapidly regain the lost weight after cessation of treatment, as seen in the above case. As this class of medication can be very expensive, loss of insurance coverage can lead to an abrupt cessation of medication. After stopping treatment, patients still need careful and continued treatment and monitoring to ensure that they do not rapidly regain the lost weight [13].

## Conclusions

As the precise pathophysiology of IIH remains unknown, the medical field continues to work to find effective treatments for this disease. Obesity is a clear risk factor for IIH, and GLP-1 receptor agonists have been some of the most effective treatments for obesity to date. They could potentially serve as another means by which to treat IIH, by promoting weight loss, lipolysis of adipose tissue, and potentially decreasing the secretion of CSF. These treatments need to be used with caution, however, as abrupt cessation of GLP-1 receptor agonists can lead to rapid weight gain in a patient who has not yet modified their lifestyle. As seen in the case presented, this rapid weight gain can result in IIH. As GLP-1 receptor agonists continue to be used in more patients, it is important for clinicians to be aware of the potential benefits of this treatment as well as potential risks, especially as the medical field works to determine how to maintain the weight loss seen with these medications long term.

## Data Availability

Not applicable.
